# Preventive Efficacy and Safety of Yiqi-Wenjing-Fang Granules on Oxaliplatin-Induced Peripheral Neuropathy: A Protocol for a Randomized, Double-Blind, Placebo-Controlled, Multicenter Trial

**DOI:** 10.1155/2021/5551568

**Published:** 2021-09-29

**Authors:** Zhancheng Gu, Guoli Wei, Liangjun Zhu, Lingjun Zhu, Jing Hu, Qi Li, Guoxiang Cai, Hong Lu, Min Liu, Chen Chen, Yi Ji, Guochun Li, Jiege Huo

**Affiliations:** ^1^The Third Clinical Medical College, Nanjing University of Chinese Medicine, Nanjing 210046, China; ^2^Department of Oncology, Jiangsu Provincial Hospital of Integrated Chinese and Western Medicine, Nanjing 210046, China; ^3^Department of Medical Oncology, Jiangsu Cancer Hospital, Nanjing 210009, China; ^4^Department of Oncology, The First Affiliated Hospital of Nanjing Medical University, Nanjing 210029, China; ^5^Department of Medical Oncology, Nanjing Drum Tower Hospital, The Affiliated Hospital of Nanjing University Medical School, Nanjing 210008, China; ^6^Department of Oncology, Shuguang Hospital, Affiliated to Shanghai University of Traditional Chinese Medicine, Shanghai 200021, China; ^7^Department of Colorectal Surgery, Fudan University Shanghai Cancer Center, Shanghai 200032, China; ^8^Department of Chemotherapy, Changshu No. 1 People's Hospital, Chuzhou 239001, China; ^9^Department of Oncology, Suzhou Hospital of Traditional Chinese Medicine, Suzhou 215002, China; ^10^Department of Oncology, Yancheng Hospital of Traditional Chinese Medicine, Yancheng 224005, China; ^11^School of Medicine and Holistic Integrative Medicine, Nanjing University of Chinese Medicine, Nanjing 210046, China

## Abstract

*Background*. Oxaliplatin-induced peripheral neuropathy (OIPN) is one of the most common side effects of oxaliplatin, which can cause reduction and cessation of oxaliplatin-based chemotherapy and significantly affect patients' quality of life. However, no drug has got recognition to prevent or treat OIPN. Yiqi-Wenjing-Fang (YWF) is a joint name of Chinese medicine prescriptions with similar effects of tonifying qi and warming meridians, represented by Huangqi Guizhi Wuwu decoction (HGWD) and Danggui Sini decoction (DSD), both from “Treatise on Cold Pathogenic and Miscellaneous Diseases.” YWF granules, including HGWD granules and DSD granules, have been, respectively, demonstrated to be effective in preventing OIPN in previous small-sample observations. The purpose of this study is to enlarge the sample size for further evaluation of the preventive efficacy and safety of YWF granules on OIPN. *Methods and Analysis.* This study is a randomized, double-blind, placebo-controlled, and multicenter clinical trial. 360 postoperative patients with stage IIa-IIIc colorectal cancer will be randomly assigned into placebo-control group, intervention group I, and intervention group II, taking the mimetic granules of YWF as placebo, HGWD granules and DSD granules, respectively. All subjects will receive oxaliplatin-based chemotherapy regimen at the same time. EORTC QLQ-CIPN20 will be used to assess the degree of OIPN as the primary outcome measure. The grades of OIPN, quality of life, chemotherapeutic efficacy, and the number of completed chemotherapy cycles are selected as the secondary outcome measures. *Discussion*. Based on the condition of no recognized effective drugs in preventing OIPN, evidence-based medical study will be conducted for seeking a breakthrough in the field of Chinese herb medicine. This protocol could provide reliable and systemic research basis about the efficacy of YWF granules and the differentiation of two classical prescriptions of YWF on preventing OIPN objectively. *Trial Registration.* This study was registered at ClinicalTrials.gov on 26 December 2020 (ID: https://clinicaltrials.gov/ct2/show/NCT04690283).

## 1. Introduction

Oxaliplatin is a prime drug in the first-line standard chemotherapy regimens of colorectal cancer, included in mFOLFOX6, FOLFOX4, XELOX, and so on. The main side effect is dose-dependent neurotoxicity, namely, oxaliplatin-induced peripheral neuropathy (OIPN), which is the primary cause of reduction and cessation of platinum-based chemotherapy. Acute OIPN usually will develop in hours of oxaliplatin infusion and peaks 3 days later. About 90% of patients will undergo acute OIPN symptoms, characterized by abnormal sensations and muscle spasm of perioral region, tongue, throat, jaw, and limbs, triggered by cold stimulation. These acute symptoms can alleviate in a week or so but will recur as the next chemotherapy cycle comes subsequently [1, 2]. Chronic OIPN will gradually develop and aggravate after approximately 3 months of repeated administration, usually with more than 540 mg/m^2^ cumulative dose of oxaliplatin. Clinical manifestations of chronic OIPN are bilateral symmetric paresthesia, dysesthesia and/or allodynia of limbs (“glove-sock” distribution), disorder of fine motor coordination, sensory ataxia, and autonomic nervous dysfunction, persisting as long as 6 months or even years after the termination of treatment [2–4].

Through exploring the mechanism of OIPN, researchers have been trying to find potential prophylactic or therapeutic drugs all the time. Numerous clinical and preclinical studies have reported a series of effective agents for OIPN, such as calcium and magnesium injections [5], carbamazepine [6], oxcarbazepine [7], venlafaxine [8], gabapentin [9], pregabalin [10], glutathione [11], glutamine [12], alpha-lipoic acid [13], amifostine [14], Vitamin E [15], monosialoganglioside (GM1) [16], and novel agents such as niclosamide [17], fingolimod [18], N-acetylcysteine [19], milnacipran [20], riluzole [21], and a selective sigma-1 receptor antagonist MR309 [22]. Nonetheless, most studies either get negative results on further validation or remain at early stages, so that seldom drug has received recognition yet [1, 23]. According to the updated American Society of Clinical Oncology (ASCO) guideline, duloxetine is the only recommended treatment agent for patients with established painful OIPN. However, limited benefits and unfavorable side effects decrease the support of duloxetine [24, 25]. Besides, some of the nondrug treatment strategies, like exercise, scrambler therapy, cryotherapy, and compression therapy, are moderately recommended for further study for their potential benefits and reasonably safety but still lack strong evidence [26–28]. Therefore, seeking effective agents for OIPN is still an urgent and significant task.

According to the clinical characteristics, OIPN corresponds with “Bizheng” from the theory of traditional Chinese medicine (TCM), interpreted as arthromyodynia. Considering the hypoimmunity of patients receiving chemotherapy, deficiency of qi and meridian impassability are regarded as the vital pathogenesis of OIPN [29]. Yiqi-Wenjing-Fang (YWF) is a joint name of TCM prescriptions with similar effects of tonifying qi and warming meridians, represented by Huangqi Guizhi Wuwu decoction (HGWD) and Danggui Sini decoction (DSD), both from “Treatise on Cold Pathogenic and Miscellaneous Diseases.” Comparing the two representative prescriptions, HGWD is better at tonifying qi, while DSD is better at warming meridians. Our previous small-sample-size randomized controlled clinical trial (*n* = 72) showed that HGWD could effectively prevent OIPN through reducing the incidence and alleviating the symptoms of OIPN without affecting the chemotherapy efficacy [30]. Further mechanistic studies preliminarily revealed the correlation between HGWD and platinum transport and accumulation in dorsal root ganglion (DRG), as well as inflammatory responses, oxidative stress, axonal lesions, and neuronal and mitochondrial dysfunctions induced by platinum chelate [31, 32]. We conducted another randomized controlled clinical trial (*n* = 48) about DSD, which showed that DSD could significantly decrease the incidence and severity of acute OIPN [33]. Mechanistic studies demonstrated that the protective effects of DSD could be related to suppressing inflammatory lesions, improving ultramicrostructures, and enhancing amounts of Nissl bodies in DRG [34]. Although YWF has a strong clinical research basis in the efficacy and safety of OIPN prevention, further studies are necessary due to the insufficient sample size and the lack of placebo and parallel controls previously.

## 2. Method and Analysis

### 2.1. Objective

YWF have been empirically used to treat arthromyodynia for a long time in China, represented by HGWD and DSD, but there is a lack of evidence-based medical study. Small-sample-size clinical studies and animal experiments have been conducted and indicated positive efficacy of YWF granules on OIPN. To enlarge the sample size for further evaluation, 360 subjects for eligibility will be recruited. In addition, in order to compare the preventive efficacy on OIPN between HGWD granules and DSD granules, two paratactic treatment groups will be set according to the theory of treatment based on syndrome differentiation.

### 2.2. Study Design

This study is designed as a randomized, double-blind, placebo-controlled, and multicenter clinical trial that will last for 18 months from January 2021 to June 2022. It will be conducted in 30 clinical centers in China, and each center will have an investigator for enrolling the patients and collecting the data. The centers and their investigators are listed in Table 1.

**Figure 1:**
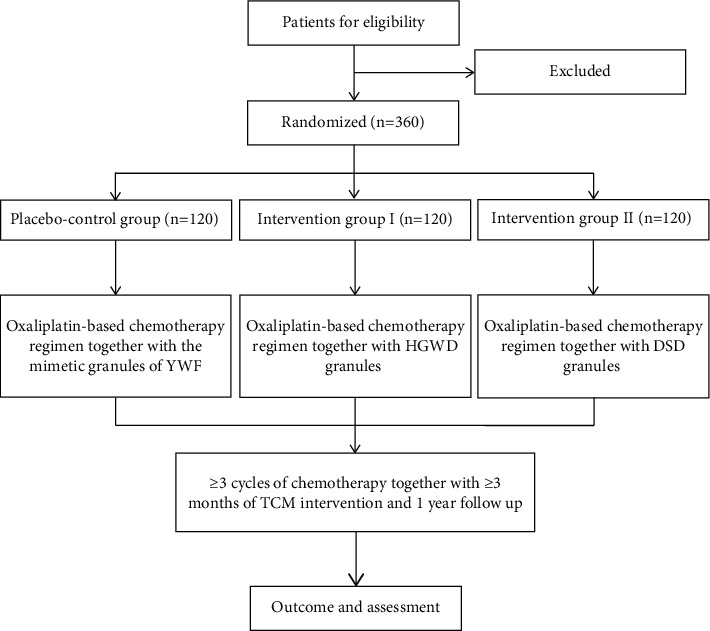
Study flow chart of enrollment, allocation, intervention, and assessment.

**Figure 2:**
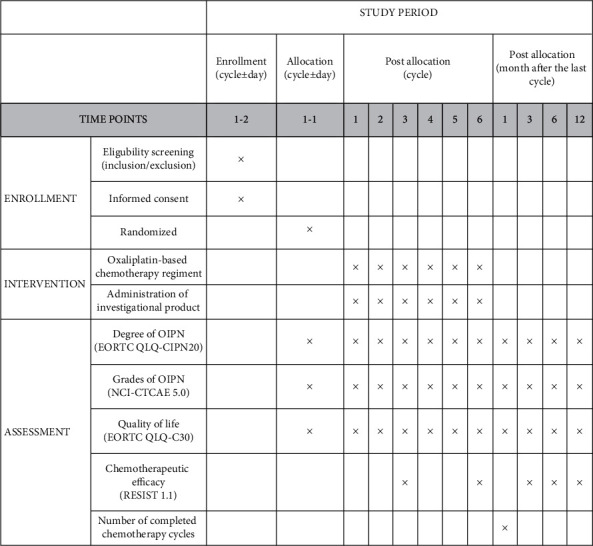
Schedule of enrollment, allocation, intervention, and assessment. *Note*. Subjects will complete the EORTC QLQ-CIPN20 questionnaire for 4 consecutive times during cycle 1 (refer to 2.8). EORTC QLQ-CIPN20: European Organization for Research and Treatment of Cancer Quality of Life Questionnaire-CIPN twenty-item scale; NCI-CTCAE 5.0: National Cancer Institute-Common Terminology Criteria for Adverse Events Version 5.0; EORTC QLQ-C30: European Organization for Research and Treatment of Cancer's Core Quality of Life Questionnaire; RECIST 1.1: Response Evaluation Criteria in Solid Tumors Version 1.1.

The trial was registered at ClinicalTrials.gov (ID: NCT04690283), and the protocol version is 1.2/20200506. The flow chart is shown in Figure 1. The schedule of enrollment, interventions, and assessments is shown in Figure 2.

### 2.3. Eligibility Criteria

#### 2.3.1. Inclusion Criteria

- Subject diagnosed with stage IIa-IIIc colorectal cancer, confirmed by histopathological examination, according to Chinese Society of Clinical Oncology (CSCO) guidelines for diagnosis and treatment of colorectal cancer.
- Subject suitable for receiving FOLFOX4 or mFOLFOX6 or XELOX as adjuvant chemotherapy after radical resection of colorectal cancer, receiving each dose and cumulative dose of oxaliplatin of 85 mg/m^2^ and ≥540 mg/m^2^, respectively.
- Subject with Karnofsky Performance Status Scale (Schag et al. 1984) index ≥60 points and an expected survival time ≥6 months.
- Subject aged between 18 and 80 years, men or women.
- Subject without severe damage of the heart, liver, kidney, or hematopoietic system.

#### 2.3.2. Exclusion Criteria

- Subject with any grade of peripheral neuropathy.
- Subject who has ever received treatment of neurotoxic chemotherapeutics, such as oxaliplatin, cisplatin, taxanes, or vinca alkaloids.
- Subject who is receiving agents with potential preventive or therapeutic effects to neuropathy, such as duloxetine, carbamazepine, venlafaxine, gabapentin, pregabalin, phenytoin, valproate, milnacipran, or tricyclic antidepressant.
- Subject who is participating or have participated in other clinical trials.
- Subject with a family history of hereditary/familial neuropathy.
- Subject who cannot take drugs orally.
- Subject with mental illness who cannot cooperate.
- Pregnant or lactation period women.

#### 2.3.3. Termination Criteria

- Subject withdraws from informed consent.
- Disease progression of subject is confirmed by imageological examination or clinical assessment.
- Subject experienced adverse events that the investigator deemed necessary to terminate trial treatment.
- Subject experienced severe side effects that the investigator deemed necessary to terminate trial treatment.
- Subject who stops or discontinues their medication without authorization.
- Subject who is less compliant with trial regulations intentionally or unintentionally.
- Other circumstances that may be detrimental to subject.

### 2.4. Sample Size Calculation

This study is suitable for superiority test. Although without positive-control group, the method of sample size calculation is equal to the three-arm trial. Based on the incidence of OIPN, the incidence of the placebo-control group was assumed as 40%, and the incidences of two intervention groups were both assumed as 20%. The Pearson chi-square test was used to calculate the appropriate sample size for each group. Taking the 20% shedding rate into account and rounding off, each group needs 120 samples and a total of 360 samples.

### 2.5. Randomization, Allocation, and Blinding

Randomization and blinding are controlled from the drug production source. All the test drugs are designed with the same appearance, dose, and smell, and each drug package has a corresponding drug number. Every six drug numbers are formed into a group, randomly corresponding to six test drugs, which necessarily include two mimetic granules of YWF, two HGWD granules, and two DSD granules, so that the whole is in accordance with the ratio of 1:1:1. Each center must recruit subjects in units of six (at least six subjects), and each recruited subject will be randomly assigned a sequential number and a corresponding drug number. Although the sequential and drug numbers are public, the specific properties of the drug are unknown to anyone, including subjects, investigators, and third-party inspectors.

### 2.6. Intervention

All subjects will receive FOLFOX4 or mFOLFOX6 or XELOX chemotherapy regimen, listed in Table 2, and YWF granules or placebo at the same time. In order to control the dosage of Chinese herbal medicine, the standard granules will be used as the intervention dosage form in this study, provided by Tianjin Hongri Kangrentang Pharmaceutical Co. LTD., China, listed in Table 3. Besides, any other drugs potentially affecting the results of the experiment will be forbidden in the whole trial process, such as ganglioside, calcium and magnesium mixture agent, glutathione, mecobalamine, and injections containing astragalus.

#### 2.6.1. Placebo-Control Group

From the beginning of chemotherapy, subjects in this group will take the mimetic granules of YWF brewed by warm water after meal twice a day (once 1 bag, 1 hour after breakfast and dinner) for at least 3 months. The mimetic granules of YWF, which contain 2.5% HGWD granules, 2.5% DSD granules, bitter principle, food colouring, and starch, are designed with the same appearance and dosage and similar smell and taste to YWF granules.

#### 2.6.2. Intervention Group I

From the beginning of chemotherapy, subjects in this group will take HGWD granules brewed by warm water after meal twice a day (once 1 bag, 1 hour after breakfast and dinner) for at least 3 months.

#### 2.6.3. Intervention Group II

From the beginning of chemotherapy, subjects in this group will take DSD granules brewed by warm water after meal twice a day (once 1 bag, 1 hour after breakfast and dinner) for at least 3 months.

### 2.7. Outcomes

In order to observe the dynamic change of OIPN severity and quality of life (QOL) to give evidence-based evaluations, this trial sets multiple measure time points.

#### 2.7.1. Primary Outcome

- The degree of oxaliplatin-induced peripheral neuropathy will be evaluated according to the score of European Organization for Research and Treatment of Cancer Quality of Life Questionnaire-CIPN twenty-item scale (EORTC QLQ-CIPN20, 20–80 scores, with higher scores meaning a worse outcome), which is evaluated by the subject on 1 day before and 3 consecutive days after the first time of oxaliplatin administration. Subsequent assessments will be made every one time from cycle 2 to cycle 6, 1 month after the end of cycle 6, and then every 3 months up to 1 year.

#### 2.7.2. Secondary Outcome

- The grades of oxaliplatin-induced peripheral neuropathy will be evaluated according to the grades of National Cancer Institute-Common Terminology Criteria for Adverse Events Version 5.0 (NCI-CTCAE 5.0, 0–5 grades, with higher grade meaning a worse outcome), which is evaluated by the doctor on 1 day before the oxaliplatin administration from cycle 1 to cycle 6, 1 month after the end of cycle 6, and then every 3 months up to 1 year.
- QOL will be evaluated according to the score of European Organization for Research and Treatment of Cancer's Core Quality of Life Questionnaire (EORTC QLQ-C30, 28–112 scores, with higher scores meaning a worse outcome) on 1 day before the oxaliplatin administration from cycle 1 to cycle 6, 1 month after the end of cycle 6, and then every 3 months up to 1 year.
- Chemotherapeutic efficacy will be evaluated according to Response Evaluation Criteria in Solid Tumors Version 1.1 (RECIST 1.1) at the end of cycle 3 (and cycle 6 if it exists).
- The number of completed chemotherapy cycles will be recorded at the end of cycle 6 or after the last chemotherapy. If the result is less then 6, the specific reason should be noted.

### 2.8. Safety Assessment

All adverse events and reactions will be recorded in detail, including the variety, occurrence time, incidence, severity, duration, therapeutic measures, and outcomes. Some examinations should be taken to monitor the adverse events and reactions, including blood routine examination, stool routine examination, liver function test, renal function test, electrocardiograph (ECG), computed tomography (CT), and magnetic resonance imaging (MRI). It should be highlighted and analyzed in particular about the abnormal inspection indicators, cases of study suspension due to adverse events, and the causality between the adverse events and the test drugs.

### 2.9. Data Management and Monitoring

For efficient and convenient statistics, this study will adopt electronic case report form (eCRF) to record data. The data inputter in each center should timely and accurately record data and upload it to the statistical system after requesting and obtaining the sequential number and the random grouping code. After being reviewed by the inspectors and checked by the data administrator, the data will be locked for statistical analysis subsequently.

This clinical trial will introduce a third-party monitoring team to strengthen the research quality control during the whole process. In accordance with the Good Clinical Practice (GCP) requirements issued by the State Food and Drug Administration (SFDA), a dedicated inspector will be assigned to make regular on-site monitoring visits to the clinical centers to ensure strict adherence to the protocol and correct filling of the test data.

### 2.10. Statistical Analysis

This study cooperates with Statistics Department of Nanjing University of Traditional Chinese Medicine. After the test trial is determined, a special statistician will be employed for making the statistical analysis plan. All data will be statistically analyzed by SAS 9.3 software. Enumeration data will be expressed as percentage, while measurement data will be expressed as mean ± standard deviation. For comparison of count or grade data, chi-square test will be used. Two logistic multiple regressions (Enter method) will be used to analyze the factors affecting the objective curative effect.

## 3. Discussion

Oxaliplatin is a standard chemotherapy drug for colorectal cancer, and its toxic and side effects must be taken seriously. OIPN is the most common side effect which significantly affects the curative efficacy of chemotherapy and patients' quality of life and remains a refractory problem despite receiving extensive attention from researchers.

For seeking potential effective therapy, the pathogenesis of OIPN has been studied for a long time. Analysis of recent literature suggested that the alteration of voltage-gated Na^+^ channels, K^+^ channels, Ca^2+^ channels, and transient receptor potential (TRP) channels could be related to acute OIPN [2]. On the other hand, platinum accumulating in dorsal root ganglion so as to cause a series of neuronal damage, including nuclear DNA damage, mitochondrial dysfunction, oxidative stress, and axonopathy, is considered as the main mechanism of chronic OIPN [2, 35]. Regarding platinum accumulation, organic cation transporter 2 (OCT2) has been demonstrated to play an important role in platinum uptake and is expected to be a new target for prevention and treatment of OIPN [36, 37].

A number of agents have been proposed with potential therapeutic or preventive effects to OIPN. However, none of them have got recognition by further studies [1, 23]. Combining the theory of TCM, this study attributes OIPN to “arthromyodynia” [29]. YWF granules are a category of prescription granules with similar effects of tonifying qi and warming meridians, and they have been empirically used to treat arthromyodynia in China. In order to obtain scientific and precise evidence, several animal studies and randomized controlled clinical trials with small sample size have been conducted previously. Results showed that YWF granules can ameliorate OIPN syndrome without affecting the antitumor activity of oxaliplatin [30, 33]. Thus, we have designed the protocol for a randomized, placebo-controlled, double-blind, multicenter trial to enlarge the sample size for further evaluation of the preventive efficacy and safety of YWF granules. This study will provide objective evidence for the clinical application of YWF granules in preventing OIPN.

## Figures and Tables

**Table 1:** Clinical centers in China.

No.	Organization name	Location	Investigator
1	Jiangsu Province Hospital on Integration of Chinese and Western Medicine	Nanjing, Jiangsu Province	Jiege Huo
2	Sir Run Run Hospital	Nanjing, Jiangsu Province	Fubing Wu
3	Yancheng Hospital of Traditional Chinese Medicine	Yancheng, Jiangsu Province	Jianlin Xu
4	Jiangning District Hospital of Traditional Chinese Medicine	Nanjing, Jiangsu Province	Zhen Wang
5	Suzhou Hospital of Traditional Chinese Medicine	Suzhou, Jiangsu Province	Min Liu
6	Huai'an Hospital of Traditional Chinese Medicine	Huai'an, Jiangsu Province	Aifei Chen
7	Zhenjiang Hospital of Chinese Traditional and Western Medicine	Zhenjiang, Jiangsu Province	Dong Fang
8	Changshu No. 1 People's Hospital	Changshu, Jiangsu Province	Hong Lu
9	Affiliated Nanjing Jiangbei People's Hospital of Nantong University	Nanjing, Jiangsu Province	Ge Feng
10	The First People's Hospital of Chuzhou	Chuzhou, Anhui Province	Aimin Chen
11	Shanghai Changzheng Hospital	Shanghai	Xiaoqiang Yue
12	Nantong Tumor Hospital	Nantong, Jiangsu Province	Chunming Xu
13	Danyang Hospital of Traditional Chinese Medicine	Danyang, Jiangsu Province	Guofang Wang
14	Nanjing Drum Tower Hospital	Nanjing, Jiangsu Province	Xiaoping Qian
15	Jiangsu Cancer Hospital, the Affiliated Cancer Hospital of Nanjing Medical University	Nanjing, Jiangsu Province	Liangjun Zhu
16	Jiangsu Province Hospital, the First Affiliated Hospital of Nanjing Medical University	Nanjing, Jiangsu Province	Lingjun Zhu
17	Fudan University Shanghai Cancer Center	Shanghai	Guoxiang Cai
18	Longhua Hospital Shanghai University of Traditional Chinese Medicine	Shanghai	Zhongqi Wang
19	Changhai Hospital	Shanghai	Xiaofeng Zhai
20	Shuguang Hospital Affiliated to Shanghai University of Traditional Chinese Medicine	Shanghai	Qi Li
21	Yangzhou Hospital of Traditional Chinese Medicine	Yangzhou, Jiangsu Province	Xiaochun Zhang
22	Zhejiang Provincial Hospital of Chinese Medicine	Hangzhou, Zhejiang Province	Qijin Shu
23	Hangzhou Hospital of Traditional Chinese Medicine	Hangzhou, Zhejiang Province	Shengyou Lin
24	The First Affiliated Hospital of Guangxi University of Chinese Medicine	Nanning, Guangxi Province	Zhen Rong
25	Henan Cancer Hospital	Zhengzhou, Henan Province	Huaimin Liu
26	Affiliated Hospital of Traditional Chinese Medicine of Southwest Medical University	Luzhou, Sichuan Province	Zhongming Yang
27	Affiliated Hospital of Shanxi University of Traditional Chinese Medicine	Xianyang, Shanxi Province	Renting Li
28	Hunan Academy of Traditional Chinese Medicine Affiliated Hospital	Changsha, Hunan Province	Puhua Zeng
29	Hebei General Hospital	Shijiazhuang, Hebei Province	Qingxia Li
30	The First Affiliated Hospital of Guizhou University of Chinese Medicine	Guiyang, Guizhou Province	Xindong Tang

*Note*. Each center was randomly assigned the center number, and the ranking had nothing to do with the center scale and the number of planned enrollment.

**Table 2:** Chemotherapy regimen.

Abbreviated name	Components	Dosage	Mode of administration	Treatment course
FOLFOX4	Oxaliplatin	85 mg/m^2^	Intravenous infusion for >2 hours on day 1	Every 2 weeks, at least 3 cycles
	Leucovorin	200 mg/m^2^	Intravenous infusion for >2 hours on days 1 and 2	
	5-Fluorouracil	400 mg/m^2^	Intravenous injection on days 1 and 2	
		600 mg/m^2^	Continuous intravenous infusion for 22 hours on days 1 and 2	

mFOLFOX6	Oxaliplatin	85 mg/m^2^	Intravenous infusion for >2 hours on day 1	Every 2 weeks, at least 3 cycles
	Leucovorin	400 mg/m^2^	Intravenous infusion for >2 hours on day 1	
	5-Fluorouracil	400 mg/m^2^	Intravenous injection on day 1	
		1200 mg/m^2^	Continuous intravenous infusion for 22 hours on days 1 and 2	

XELOX	Oxaliplatin	130 mg/m^2^	Intravenous infusion for >2 hours on day 1	Every 3 weeks, at least 4 cycles
	Capecitabine	1000 mg/m^2^	Oral administration twice a day on days 1–14	

**Table 3:** Standard formulation of YWF granules.

Huangqi Guizhi Wuwu decocion (HGWD) granules			
Pinyin name	English name	Latin name	Doses, g
Huangqi	*Astragalus membranaceus*	Astragali Radix Membranacei	18
Guizhi	Cassia twig	Ramulus Cinnamomi	9
Baishao	Radix Paeoniae Alba	Paeoniae Radix Alba	9
Shengjiang	Ginger	Rhizoma Zingiberis Recens	9
Dazao	Jujube	Fructus Zizyphi Jujubae	9

Danggui Sini decoction (DSD) granules			
Danggui	*Angelica sinensis*	*Angelicae sinensis*	12
Guizhi	Cassia twig	Ramulus Cinnamomi	9
Baishao	Radix Paeoniae alba	Paeoniae Radix Alba	9
Xixin	*Asarum*	Herba Asari Cum Radice	3
Tongcao	Tetrapanax papyrifer	Medulla Tetrapanacis Papyriferi	6
Zhigancao	Honey-fried licorice root	Radix Glycyrrhizae Preparata	5
Dazao	Jujube	Fructus Zizyphi Jujubae	9

## Data Availability

The data and materials are available upon request from the corresponding author.
